# Seeing the World From Others’ Perspective: 14-Month-Olds Show Altercentric Modulation Effects by Others’ Beliefs

**DOI:** 10.1162/opmi_a_00050

**Published:** 2022-02-09

**Authors:** Dora Kampis, Ágnes Melinda Kovács

**Affiliations:** Department of Psychology, University of Copenhagen, Denmark; Department of Cognitive Science, Central European University, Budapest, Hungary/Vienna, Austria

**Keywords:** altercentrism, infants, manual search, object representation, theory of mind, aspectuality

## Abstract

Humans have a propensity to readily adopt others’ perspective, which often influences their behavior even when it seemingly should not. This altercentric influence has been widely studied in adults, yet we lack an understanding of its ontogenetic origins. The current studies investigated whether 14-month-olds’ search in a box for potential objects is modulated by another person’s belief about the box’s content. We varied the person’s potential belief such that in her presence/absence an object was removed, added, or exchanged for another, leading to her true/false belief about the object’s presence (Experiment 1, *n* = 96); or transformed into another object, leading to her true/false belief about the object’s identity (i.e., the objects represented under a specific aspect, Experiment 2, *n* = 32). Infants searched longer if the other person believed that an object remained in the box, showing an altercentric influence early in development. These results suggest that infants spontaneously represent others’ beliefs involving multiple objects and raise the possibility that infants can appreciate that others encode the world under a unique aspect.

## INTRODUCTION

The highly social nature of human existence requires us to be equipped with cognitive mechanisms that allow proficient navigation in the social world. As individuals tend to differ in their knowledge, intentions, and abilities, we often have to rapidly infer others’ unique perspective. Various abilities may support human sociality, some of which, for example, sensitivity to others’ beliefs, may be present early in ontogeny (Baillargeon et al., 2010). Uncovering the nature and development of these cognitive mechanisms is still one of the liveliest domains in research on social cognition (Baillargeon et al., 2018a; Carruthers, 2013; Perner & Ruffman, 2005; Poulin-Dubois et al., 2018; Rakoczy, 2012). It is uncontroversial that humans eventually arrive to the ability to consider others’ mental states when they are prompted to, for example in communicative or collaborative contexts (Hanna et al., 2003; Schober, 1993). However, considering what others see or know can also be cognitively challenging, making us *egocentric*: attending to our own perspective and only with effort attend to, or judge, that of others (Keysar et al., 2003; Nickerson, 1999). In line with humans’ pronounced sociality compared to other animals (Herrmann et al., 2007), however, recently it has been argued that humans may in fact have a strong tendency to attend to others (Kovács et al., 2010). People often seem to readily compute others’ perspective and mental states—that is, what and how they perceive in their environment and the consequent mental representations they may form—even when this is not required by the situation or task they are performing, or it is even disadvantageous. Humans are thus inclined to spontaneously focus on the perspective of others: they tend to be *altercentric* (Samson et al., 2010), and a growing body evidence suggests that this tendency is more widespread than previously considered (Kampis & Southgate, 2020).

Altercentrism in human cognition reflects that people’s behavior is spontaneously influenced by the presence, and perspective, of others, even when their task requires them to focus on their own point of view (Elekes et al., 2017; Kovács et al., 2010; Samson et al., 2010). For example, adults typically detect a ball slower that appears from behind an occluder when they do not expect it to be present. However, they detect the ball slightly faster when another agent in the scene (who is not relevant for participants’ task) believes it to be present, indicating an influence of the agent’s belief on people’s responses (Kovács et al., 2010). While a subsequent study proposed that this effect may have been due to a manipulation in the paradigm related to the timing of participants’ button presses during attention checks (Phillips et al., 2015), others have adapted the paradigm with matched timings between the different trials, replicated the effect, and consequently ruled out the attention check-based alternative explanation (el Kaddouri et al., 2019). The sensitivity to the other’s perspective also appears in joint tasks where participants act together (Elekes, Bródy, et al., 2016; Freundlieb et al., 2016). While one likely role of altercentrism is facilitating interactions, others’ perspective can influence people’s behavior even when there is no obvious or immediate interactive context. Even if the other is not engaged in any task, adults’ reaction times are affected by the belief of another agent (Kovács et al., 2010; Van Der Wel et al., 2014), or by a mismatch between their own and someone else’s perspective (Samson et al., 2010). From a broader perspective, altercentrism in adults has been argued to enable rapid computations of others’ mental states (Kovács et al., 2010), likely supporting human social behavior in general. If altercentric effects are indeed as widespread in humans as indicated by recent accounts (Kampis & Southgate, 2020), this raises the question whether and how they manifest in ontogeny.

Early in development, a large body of studies points toward infants’ sensitivity to others’ visual and mental perspective (Baillargeon et al., 2010; Carruthers, 2013; Kampis et al., 2020). Besides direct and conceptual replications of various paradigms used with infants (Buttelmann et al., 2015; Király et al., 2018; Schneider et al., 2012; Thoermer et al., 2012), there has also been a recent increase in studies unable to replicate some of the original findings (Baillargeon et al., 2018b; Barone et al., 2019; Kulke & Rakoczy, 2018; Poulin-Dubois et al., 2018), leading to a still ongoing debate regarding the replicability of these phenomena, and giving rise to multi-lab collaborations aiming at large-scale studies probing infants’ performance on different theory of mind tasks (Schuwerk, Kampis, et al., 2021). At the same time, the underlying abilities have been heavily debated, some accounts arguing that evidence on infants’ mental state understanding can be explained by cognitive mechanisms that do not fully resemble those present in older children or adults (Apperly & Butterfill, 2009; Butterfill & Apperly, 2013), or do not pertain to mental state reasoning at all but are rather explained by perceptual biases (Heyes, 2014) or rule-based heuristics (Perner & Ruffman, 2005). Relatedly, it has been a long-held tradition that children start out egocentric, based on findings showing that children have difficulties thinking about the perspective of others (Piaget, 1926), to the extent that similarly to adults they experience interference from their own knowledge (Birch & Bloom, 2004). In contrast, Southgate (2020) recently proposed that from a developmental perspective it could be particularly advantageous to have a disposition to attend to others’ point of view from early on, arguing that young infants may be altercentric and show pronounced attention to others’ perspective.

Altercentrism may indeed have a broader epistemic function by allowing us to identify what information others possess when we aim to seek or transmit information. Infants face a crucial learning task when discovering the world, and the social environment can help with identifying relevant sources of information and guide their leaning. Attention to others’ perspective may enable infants to learn about the material world via the perspective of others around them. For instance, studies have suggested that infants may learn about the valence of objects through the preferences of others (Egyed et al., 2013; Kampis et al., 2013), and map a new word to an object that was in the speaker’s center of attention, and not what they themselves were attending to (Baldwin, 1991). Consequently, one might wonder whether infants, similarly to adults, may also show altercentrism. There is neuroimaging evidence that is consistent with this possibility. Some work indicates that infants’ own representations and those used to encode others’ perspective are handled at least in part by the same system (Kampis et al., 2015; Southgate & Vernetti, 2014), potentially increasing commensurability between the two and providing grounds for explanation on how the other’s perspective may influence infants’ behavior. Crucially, while there are studies documenting altercentric effects in children (Buttelmann & Buttelmann, 2017; Elekes et al., 2017; Milward et al., 2014), to our knowledge there is only one study that speaks to altercentric modulation in infants, where infants’ looking times were influenced by another agent’s belief (Kovács et al., 2010).

The current studies asked whether altercentric modulation can be found in 14-month-old infants in an active behavioral task. We adapted a task in which infants search longer in a box if they think an object is still inside, compared to when the box is empty (Feigenson & Carey, 2003). The target age group was chosen as it is from this age onward when the effects of conceptual and social information on infants’ behavior have been reliably probed with this paradigm (Feigenson & Carey, 2003; Feigenson & Halberda, 2008; Stahl & Feigenson, 2014, 2018). Our main goal was to establish the existence of altercentric effect in preverbal infants in a task involving an active behavioral response. We modified the manual search paradigm to assess how long infants would search depending *on another person’s belief* (i.e., the belief infants may ascribe to this person). Specifically, we asked whether infants search longer in a box if the other person believes that it contains an object, compared to when she believes it is empty, while infants’ own belief was kept constant. In Experiment 1, to track the other’s belief, infants had to keep in mind how many objects she believed to be in the box. In Experiment 2, tracking the other’s belief required identifying the unique perspective or aspect (Perner et al., 2011) under which she represented these objects.

## EXPERIMENT 1

### Method

In the three conditions of Experiment 1 we assessed whether 14-month-old infants’ (*n* = 32/condition) track events involving multiple objects and ascribe to others beliefs based on spatiotemporal or feature-based tracking; and whether representing others’ beliefs may modulate infants’ own search behavior. The conditions differed in the events that led to the Actor’s belief: whether in her presence or absence an object was (i) removed from-, (ii) added to-, (iii) or exchanged in the box, corresponding to the three conditions. All studies received full ethical approval from the United Ethical Review Committee for Research in Psychology in Hungary and were conducted according to the principles of the Declaration of Helsinki. Participants’ caregivers gave written informed consent prior to participation. Datasets of the studies are available at the OPMI Dataverse: https://doi.org/10.7910/DVN/ZVEBZH. Video recordings cannot be made publicly available due to privacy restrictions; sample videos are provided in the Supplemental Materials.

### Participants

Ninety-six healthy full-term 14-month-old infants participated (age range from 14;0 [Months; Days] to 15;1, mean age = 14;17) in the study, randomly assigned to one of the three conditions (*N* = 32/condition). The number of infants per condition was based on the study of Feigenson and Carey (2003) who included *n* = 32 infants, and a power analysis for the main analysis (one-sample *t* test on the difference scores) with medium effect size of Cohen’s *d* = 0.5, a power of 0.8 and alpha of 0.05, yielding *n* = 34. We therefore included *n* = 32 per condition, yielding a total of *n* = 96. Thirty-seven additional infants were tested but not included in the analyses because of passivity (they did not search in any of the trials, 22), or because the study was not completed because the baby fussed out (10), or due to parental interference or experimental error (in the procedure or failure of the recording system) (5). These inclusion rates are similar to those in previous studies using the manual search paradigm (Feigenson & Carey, 2003).

### Procedure

We adapted a manual search task that was previously developed to study how infants track objects and perform simple arithmetic operations on them from their own, first-person perspective (Van de Walle et al., 2000). In such a task (Feigenson & Carey, 2003), 14-month-olds witnessed a certain amount (2, 3, or 4) of objects placed inside an opaque box. Next, infants were allowed to retrieve some of the objects, and then were allowed to search further. Measuring the duration of infants’ manual search in the box showed in such studies that infants searched more persistently in the box if one of the objects was still inside, compared to when all objects were already retrieved.

In the present experiments, in each condition infants first received three familiarization trials that introduced them to the objects and the setup. In these, the experimenter (who later served as Actor, referred to as A), first hid an object in the box and then searched for it and retrieved it herself, then in two subsequent trials she hid another object and encouraged the infant to search for it. The purpose of these trials was to show the infants that objects can be retrieved from the box, as well as to introduce an interaction between the Actor and the child via a hiding and retrieving game, where his or her perspective about the presence or absence of the objects could become salient. The other experimenter (the Confederate: C) sat passively during these trials (see the Supplemental Materials methods for further details on familiarization and test trial procedure).

Familiarization trials were followed by two test trials where the other person’s belief was varied between trials. Within each condition infants received two test trials (in counterbalanced order, randomly assigned): one trial where the Actor (depending on condition, correctly or falsely) believed an object to be in the box [Actor believes: object present (A_obj_pres_) trials] and one where she (correctly or falsely) believed it was absent [Actor believes: object absent (A_obj_abs_) trials], while the infant’s belief was constant across the two trials.

**Figure 1.**
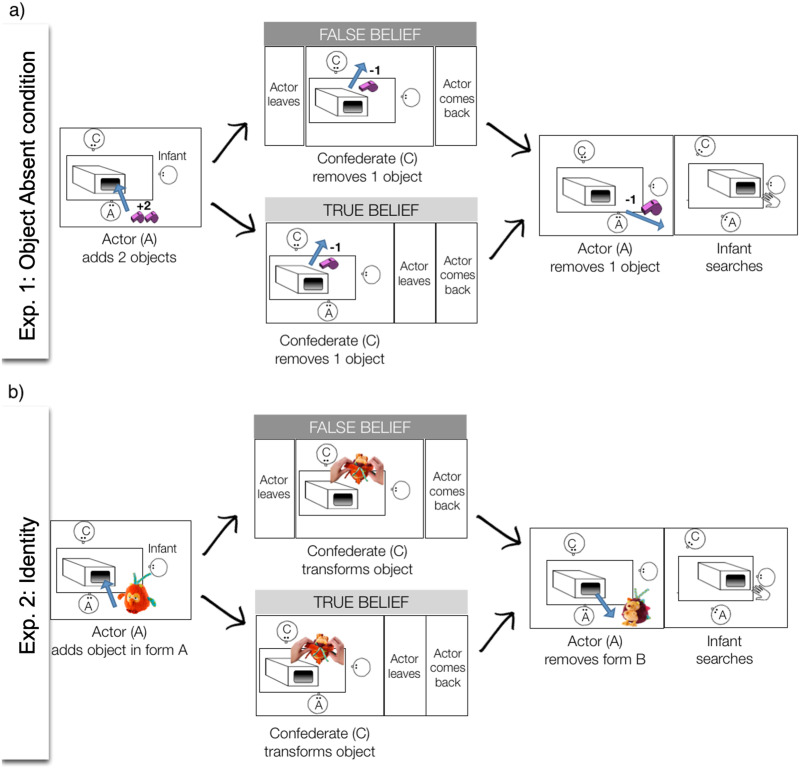
**Schematic depiction of events in test trials of a) Experiment 1: Object Absent condition, and b) Experiment 2.** In Experiment 1 the other two other conditions differed from the Object Absent condition in the following ways. In the Object Present condition, the Actor (A) added initially only one object, and in A’s presence/absence the Confederate (C) added one more object. In the Object Exchanged-Absent condition A initially added one object, and in her presence/absence C exchanged this object to an object of a different kind. All three conditions ended with A removing one object, followed by a 15-s period during which infants were allowed to search in the box. For further details on the procedure see text and Supplemental Materials.

In all conditions of Experiment 1, in the beginning of the test trials it was A who took out the object(s) from a purse on her side and put them in the box. Then, A’s belief was varied depending on when she left the room (see Figure 1) and the events she has witnessed: she either left right after the hiding, or she stayed on while C added/retrieved/exchanged an object (depending on condition, see below) and left after that. In all test trials upon her return A retrieved one object from the box, and then the 15-s measurement period followed. During the measurement period infants had the opportunity to search in the box, after which the experimenter retrieved any “remaining” objects from the box before the next trial. Importantly, in order to avoid the confound of some infants finding the object faster by chance, in all cases when an object had “remained” in the box, this object was in fact surreptitiously put into a hidden compartment. Therefore, infants did not find an object in any of the test trials in Experiment 1 or 2, thus search durations reflect their persistence to search for a (potential) remaining object.

In the *Object Absent condition* initially there were two objects in the box, then both were sequentially removed, therefore at the end of the trial infants knew the box was empty. In one of two test trials the Actor (A) did not witness that the second object was removed by the Confederate (C), and therefore believed that it remained in the box (Actor believes object present, A_obj_pres_; false belief trial). In the other test trial both A and the infant knew that all objects had been retrieved (Actor believes object absent, A_obj_abs_; true belief trial); see Figure 1.

In the *Object Present condition* there was initially only one object in the box, then a second (identical) one was added by C. Finally, one of the two objects were removed, thus in the end one object remained in the box. Infants in this condition therefore expected an object to remain in the box. In one of two trials A did not see that a second one was added and therefore in the end believed the box was empty (Actor believes object absent, A_obj_abs_; false belief trial). In the other trial A knew the second object was added and thus also expected an object to remain in the box (Actor believes object present, A_obj_pres_; true belief trial).

In the *Object Exchanged-Absent condition* initially there was one object in the box, which was then exchanged to another (differently looking) one by C. Finally, this second object was removed, therefore at the end of the trial infants knew the box was empty. In one of two trials A did not witness that the first object was exchanged to the second, and therefore believed that the first object remained in the box by the end of the trial (Actor believes object present, A_obj_pres_; false belief trial). In the other test trial both A and the infant knew that when the second object was removed the box remained empty (Actor believes object absent, A_obj_abs_; true belief trial).

### Materials

We used a white cardboard box (29*29*15 cm) with a 14*8 cm opening that was covered by an elastic cloth that prevented infants from seeing inside the box but enabled reaching into it. The box had a hidden compartment in its back where objects could be hidden, and that was inaccessible to the infants. In familiarization we used colorful whistles with a ball inside that made a rattling sound. In test there were whistles of different colors (red, green, or blue) that did not rattle, and an additional toy rattle in the Object exchange condition (see the Supplemental Materials, Figure S1a–c).

### Coding

We coded the duration of infants’ searching during the 15-s period when the box was in front of them. Infants were scored as searching whenever one or both hands were in the box, with their knuckles passing the entrance cloth of the box. If infants reached into the box opening, but clearly just manipulated/played with the cloth, we did not count it as searching. Half of the participants (*n* = 16) of each condition (Object Absent, Object Present, and Object Exchanged-Absent) were coded on both trials by a second coder who was unaware of the purpose of the study and the trial type (A_obj_abs_ vs. A_obj_pres_); interrater agreement was *r*(96) = .972, *p* < .001.

We calculated an altercentric modulation score for each infant via subtracting their search duration on A_obj_abs_ trial (A believed there was no object in the box) from their search duration on the A_obj_pres_ trial (A believed there was still an object in the box). Thus, the altercentric modulation score reflected the difference in search times within participants, between the two trials of a condition. Such a difference score is commonly used in this type of task, as absolute search times may vary across infants (Feigenson & Carey, 2003; Stahl & Feigenson, 2018). A modulation score above 0 would reflect that infants search longer when the other believes the object to be present, compared to when he or she believes the box is empty, given that the infants’ own beliefs were kept constant.

### Results

**Figure 2.**
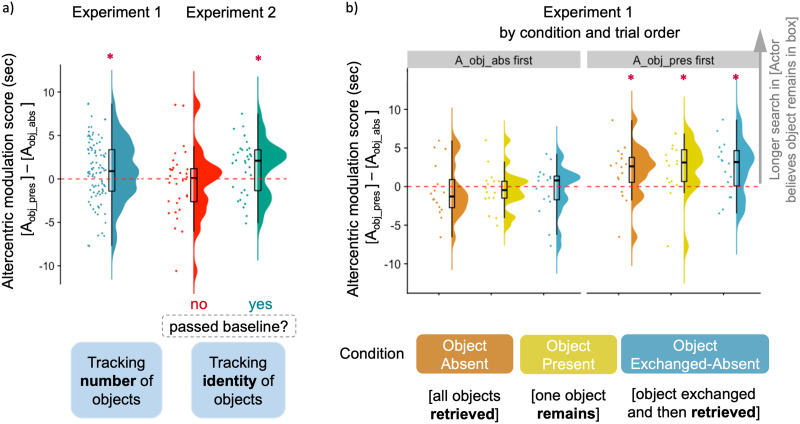
**Altercentric modulation scores (difference in search durations between A_obj_pres_: Actor believes object is present and A_obj_abs_: Actor believes no object remained trials) in a) Experiment 1 (*N* = 96); and Experiment 2 (*N* = 64) split by whether infants passed the criterion in baseline trials, and in b) Experiment 1 split by order of test trial.** Boxes indicate interquartile range with median, whiskers show 1.5 times the interquartile range, and dots show individual data points (horizontally jittered) (modified from (Allen et al., 2018). Asterisks indicate significant difference from zero.

We first compared the altercentric modulation scores in Experiment 1 to chance level (0) (for mean search values on individual trials, see the Supplemental Materials, Figure 4). Infants on average searched longer on A_obj_pres_ trials than on A_obj_abs_ trials, as reflected by a positive altercentric modulation score (*M*_*diff*_ = 0.924 s, *SD* = 3.529; see Figure 2a) significantly above chance, *t*(95) = 2.565, *p* = .012; Cohen’s *d* = 0.262, 95% CI = [0.208, 1.639] (all statistical tests two-tailed). Thus, infants overall searched significantly longer when the Actor believed an object remained in the box, compared to when she believed there was no object left.

Subsequently the altercentric modulation scores were entered into a univariate ANOVA with condition (Object Absent / Object Present / Object Exchanged-Absent) and Order of Test (A_obj_pres_ first or A_obj_abs_ first) as between-subject factors, yielding a significant effect of Order of Test, *F*(1, 90) = 15.59, *p* < .001, *η*_*p*_^2^ = .148, no effect of condition, *F*(2, 90) = 0.099, *p* = .906, *η*_*p*_^2^ = .002, and no interaction between condition and order, *F*(2, 90) = 0.032, *p* = .969, *η*_*p*_^2^ = .001. The altercentric modulation score was different from chance in the A_obj_pres_ first order, *t*(47) = 4.581, *p* < .001; Cohen’s *d* = 0.661, *M*_*diff_A_obj_pres_first*_ = 2.271 s, *SD* = 3.434, 95% CI = [1.274, 3.268], but not in the A_obj_abs_ first order, *t*(47) = .943, *p* = .35; Cohen’s *d* = 0.136, 95%, *M*_*diff_A_obj_abs_first*_ = −0.423 s, *SD* = 3.109, CI = [−1.326, 0.479]. Shapiro-Wilk tests showed no significant departure from normality, either on the entire sample, W(96) = 0.988, *p* = .574, or on the two groups split by trial order, W(48) = 0.963, *p* = .131 and W(48) = 0.984, *p* = .731 in A_obj_pres_ first and A_obj_abs_ first, respectively.

The effect of order on the altercentric modulation scores (i.e., on the difference between trials) indicates that the two belief trials were significantly different from each other in one order and not in another. To check whether the data pattern holds even when trial order cannot play a role, we analyzed search times on the first trials between participants. A Shapiro-Wilk test showed no significant departure from normality, W(96) = 0.982, *p* = .217. A univariate ANOVA with condition (Object Absent / Object Present / Object Exchanged-Absent) and Type of first trial (A_obj_pres_ first or A_obj_abs_ first) as between-subject factors showed an effect of Type of first trial, *F*(1, 90) = 4.901, *p* = .029, *η*_*p*_^2^ = .052. Infants who received an A_obj_pres_ trial (Actor believes object present) on the very first trial searched significantly longer than those receiving an A_obj_abs_ trial (Actor believes object absent) on the first trial, indicating that the effect of the other’s belief on infants’ search manifested also when trial order could not play a role. Mean raw search scores on the first trials were *M*_*A_obj_pres*_ = 6.490 s, *SD* = 3.026 and *M*_*A_obj_abs*_ = 5.042 s, *SD* = 3.374. There was no effect of condition, *F*(2, 90) = 0.228, *p* = .797, *η*_*p*_^2^ = .005, and no interaction between type of trial and condition, *F*(2, 90) = 1.786, *p* = .174, *η*_*p*_^2^ = .038.

### Discussion

Infants searched longer when according to the Actor’s belief an object remained in the box, compared to when she believed all had been retrieved. As infants’ belief about the box’s contents was constant within the trial pairs, this difference arguably reflects the effect of the Actor’s belief on infants’ behavior. The difference in modulation scores in the two presentation orders could arise from the first trial being more sensitive to factors increasing search times (e.g., the Actor’s belief in A_obj_pres_), combined with children being less easily motivated to search later on. Thus, two different effects may be working against each other: a decrease across trials in motivation to search (Van de Walle et al., 2000) and an effect of the other’s belief on infants’ search. The influence of the other’s belief on infants’ search times also holds in a between-subject analysis of the first trials, confirming that the main effect stems from our conceptual manipulation even when trial order cannot play a role.

Overall, these results show that infants tracked another person’s beliefs involving individuation and tracking of multiple objects based on spatiotemporal and feature information. Experiment 2 was set out to investigate whether infants ascribe to others’ beliefs involving aspectuality (Perner et al., 2011)—that is, whether they are sensitive to the unique aspect under which the other represents the objects.

## EXPERIMENT 2

Whether infants are sensitive to the aspectuality of beliefs is of importance because a fully developed theory of mind entails the ability to appreciate that belief representations reflect some (but not all) aspects of the environment, from the observer’s own perspective (Perner et al., 2011). As objects may have several distinct characteristics (e.g., a toy can be red, but also make a rattling sound), which of these characteristics one is aware of will guide their inferences regarding that object. Understanding others’ beliefs involving aspectuality has been proposed to require complex representational abilities that emerge after the age of four, therefore such representations should posit a fundamental challenge to the infant mind (Butterfill & Apperly, 2013; Rakoczy, 2017).

While recent evidence suggests at least by 18 months of age infants might successfully track belief scenarios involving some forms of aspectuality (Buttelmann et al., 2015; Fizke et al., 2017; Scott & Baillargeon, 2009), it is an open question whether infants grasp a fundamental inferential implication of understanding aspectuality: that another person’s partial information about an object can lead her to be mistaken about the identity of objects, and posit the existence of the two objects instead of one. We tested whether 14-month-old infants understand beliefs relying on the person’s mistaken individuation of a single object with two different aspects as two (thus holding a false belief about its identity), based on the aspect (here: visual appearance) under which she perceives them. In first-person computations a majority of 14-month-old infants could override their default assumption that different appearances signal distinct objects, if they were shown that objects could have dual appearances (Cacchione et al., 2013). Experiment 2 asked whether infants can use such inferences to represent another person’s belief about object identity. Considering that infants’ limitation to inferentially individuate objects from their own perspective may constrain them in attributing such contents to others (in Cacchione et al., 2013, approximately 70% of infants showed the predicted behavior), we introduced two baseline trials to assess whether infants individuate objects on the basis of their knowledge about the two appearances, and predicted that infants who show this understanding would also successfully track similar events from the other person’s perspective, and show altercentric modulation in belief trials.

### Methods

Infants were first presented with two belief trials in counterbalanced order (randomly assigned): true belief (A_obj_abs_) and false belief (A_obj_pres_) trials. Following this, they received two baseline trials in fixed order: an unknown-transform (B:I_obj_pres_) trial, then a known-transform (B:I_obj_abs_) trial. The fixed order of baseline trials was implemented in order to avoid carryover from known-transform (B:I_obj_abs_) trials (i.e., that infants would assume that all objects may transform).

### Participants

Sixty-four 14-month-old infants participated (age range from 14;2 to 15;3 mean age = 14;16). Thirty-five additional infants were tested but not included in the analyses because of passivity—they did not search in either of the belief test trials (13), they fussed out (15), or due to parental interference or experimental error in the procedure (7). Sample size was determined as for Study 1. We aimed to have *n* = 32 infants who passed the baseline criterion as we expected, specifically those infants to track the other’s belief and thus show an effect in the belief trials.

### Materials

We used a white cardboard box as in Experiment 1, but slightly larger in order to have a large enough opening for the new objects (that were slightly bigger than in Experiment 1). As experimental objects we used plush figures that could change their appearance via turning them inside out. We used toys of somewhat different size, color, and material in baseline and test trials, and in each individual trial a new toy was used that differed in appearance/category, to minimize the risk that infants would assume that A is knowledgeable about the objects in subsequent trials (for details see the Supplemental Materials).

### Procedure

#### Familiarization Trials.

The first two familiarization trials were identical to Experiment 1, but we introduced a third familiarization that came after the two belief test trials and served to reengage infants before the baseline trials.

#### Test Trials.

In Experiment 2 in test trials, it was always the Confederate (C) who took out the objects from her bag, to avoid any suspicion children might have that the Actor (A) has knowledge of the transforming feature of the objects. C took out an object, put it on the top of the box, and named it. Then A repeated the label and put the object into the box. In A_obj_pres_ trials, A left at this point. Then C retrieved the toy and demonstrated the transformation (while labeling both appearances with different labels), and then put the object in its new form back into the box. In A_obj_abs_ trials, A left at this point. Then in both trials A came back, sat down and retrieved the toy in its new form, and put the toy away into a bag, followed by a measurement period. In the end of the trials, C always demonstrated the reverse transformation when A was present. After this the second test trial followed. Thus, in A_obj_abs_ trials, A knew there was only one object with two identities, which was now retrieved, leaving the box empty; while in A_obj_pres_ trials A must have thought there were two objects, one of which likely still remained in the box.

#### Baseline Trials.

##### Unknown Transform (B:I_obj_pres_) trial.

In baseline trials, C sat passively at the table. A took out an object from her bag, named it, then put the object into the box, and then retrieved it in its changed form (as the transformation was done invisibly to the child, inside the box), and then put it away. Then a measurement period followed. If infants individuated two different objects from the two appearances, they should search longer for the “remaining” one (while, in fact, the box was empty).

##### Known Transform (B:I_obj_abs_) trial.

In these trials, A did not do the transformation surreptitiously inside the box, but retrieved the toy, showed the transformation, and put it back. Thus, if infants followed the transformation, indicating that the two appearances belong to the same object, when she later retrieved it in the new form, they knew that the box was emptied.

### Coding

Search durations were coded as in Experiment 1. Twenty-five percent of infants (*n* = 16) were coded on all four trials (unknown transform vs. known transform baseline trials, and A_obj_abs_ vs. A_obj_pres_ test trials) by a second coder who was unaware of the purpose of the study; interrater agreement was *r*(64) = .951, *p* < .001.

To determine whether infants made the predicted first-person inferences in baseline (that is, whether they assume that one object is still present in the box when not seeing the transformation), we set a criterion that aimed to grasp a meaningful difference between search durations in the two baseline trials. Infants were considered to have “passed” the baseline criterion if search duration on the unknown transform trials was equal or higher than the search duration on the known transform trial multiplied by the constant of 1.2, which was based on the average ratio between search times in the two types of trials in Experiment 1. If infants did not search at all in the known-transform baseline trial (which is considered a valid response), since a value of zero cannot be multiplied to produce a meaningful minimum value for the unknown transform trial, a minimum of 500-ms search duration in the unknown-transform baseline was set to categorize an infant as “passer” (see details in the Supplemental Materials).^1^

### Results

We aimed at 32 infants who qualify as passing this baseline criterion, and 32 additional infants finished the experiment but did not pass baseline. In accordance with our criterion, in the passing group infants searched longer on B:I_obj_pres_ trials than in B:I_obj_abs_ trials (*M*_B:I_obj_pres_ = 5.224 s, *SD* = 4.056; *M*_B:I_obj_abs_ = 1.577 s, *SD* = 1.904). This was not the case in the “nonpassing” group, where infants in fact searched longer on B:I_obj_abs_ trials than on B:I_obj_pres_ trials (*M*_B:I_obj_pres_ = 2.419 s, *SD* = 3.843; *M*_B:I_obj_abs_ = 3.361 s, *SD* = 4.207).

To test the prediction that particularly those infants who show evidence of individuating objects on the basis of their own knowledge about the two appearances of an object in the baseline trials, would also show an altercentric modulation effect taking into account the aspect under which A saw the object; for the main analysis we analyzed infants’ search times who have passed the baseline criterion. In the group who passed baseline (*n* = 32), infants searched on average longer on A_obj_pres_ trials than on A_obj_abs_ trials (*M*_*diff_passing*_ = 1.345 s, *SD* = 3.208; see Figure 2); and a one-sample *t* test showed that this modulation score was significantly different from chance, *t*(31) = 2.371, *p* = .024; Cohen’s *d* = 0.419, 95% CI = [0.188, 2.501]. We then split infants based on the order of test trial and compared modulation scores in the two test orders (A_obj_pres_ first vs. A_obj_abs_ first), *t*(30) = 1.914, *p* = .065; Cohen’s *d* = 0.676, 95%, *M*_*A_obj_pres_first*_ = 2.386, *SD* = 2.76, *M*_*A_obj_abs_first*_ = .303, *SD* = 3.363, 95% CI = [−0.139, 4.305]; see the Supplemental Materials for further analyses and raw search times.

To compare the number of infants who show the altercentric modulation effect in the passing baseline and the nonpassing groups, we applied the same criterion for the test trials as to the baseline trials and categorized the entire sample of infants in Experiment 2 into “shows altercentric effect” in test and “no effect” in test. Infants who searched 1.2 times longer in A_obj_pres_ than in A_obj_abs_ test trial, or at least 500 ms in A_obj_pres_ if they didn’t search in A_obj_abs_, were categorized as “shows altercentric effect.” A chi-square test showed that significantly more infants were categorized as “shows altercentric effect” in the passing baseline group, *χ*^2^(1, *N* = 64) = 5.067, *p* = .045. Thus, applying the same criteria in test trials as in baseline trials indicated a significant relation between passing baseline and showing an altercentric effect.

Additionally, to provide convergent evidence that infants’ performance on baseline was related to the altercentric modulation in test trials, we correlated difference scores from baseline trials with the altercentric modulation scores from test trials, which revealed a significant positive correlation between the two (*r* = .258, *n* = 64, *p* = .04). Together, these results give us confidence that while the passing baseline criterion is novel as it was determined for the purpose of this study, it is a reliable indicator of whether infants demonstrated an ability of understanding the dual identity objects and the scenario from their first-person perspective, which in turn was related to how they processed the scenarios taking into account the perspective of another person and the aspect under which he or she represented these objects.

As passing baseline as a criterion served to select a group of infants who do understand and track the events from their own perspective, one might wish to be cautious to make claims about the nonpassing group’s potential inability to do so. Nevertheless, as it may be informative for future research, we analyzed infants’ behavior in the nonpassing group as well. In the nonpassing group the altercentric modulation score was not significantly different from chance, *M*_*diff_nonpassing*_ = −0.428 s, *SD* = 3.927, *t*(31) = 0.617, *p* = .542; Cohen’s *d* = 0.109, 95% CI = [−1.844, 0.987], and an independent-samples *t* test comparing the two test orders (A_obj_pres_ first vs. A_obj_abs_ first) showed no difference, *t*(30) = 1.160, *p* = .255; Cohen’s *d* = 0.402, *M*_*A_obj_pres_first*_ = .424, *SD* = 4.889, *M*_*A_obj_abs_first*_ = −1.181, *SD* = 2.77, 95% CI = [−1.221, 4.429]. Shapiro-Wilk tests showed no significant departure from normality, either in the baseline passing group, W(32) = 0.964, *p* = .345, or in the nonpassing group, W(32) = 0.958, *p* = .246. We then analyzed altercentric modulation in a univariate ANOVA with passing baseline (passing / nonpassing) as between-subject factors. Altercentric modulation scores were *M*_*diff_passing*_ = 1.345 s (*SD* = 3.208) in the passing baseline group and *M*_*diff_nonpassing*_ = −0.428 s (*SD* = 3.927) in the nonpassing, effect of passing was *F*(1, 64) = 3.913, *p* = .052, *η*_*p*_^2^ = .059.

In sum, we identified infants who varied their searching behavior depending on their *own* knowledge about the object’s identity in the respective baseline trials, thus demonstrating evidence of tracking the events from their first-person perspective. Crucially, infants in this baseline-passing group searched longer in the box when according to the other person’s (false) belief there were two separate objects, and after the removal of an object she believed that one object remained in the box (A_obj_pres_ trials); compared to when she knew that there is only one object with two appearances, and therefore believed that in the end all objects have been retrieved (A_obj_abs_ trials).

### Discussion

Experiment 2 showed that 14-month-old infants who are sensitive to the dual identity of objects in first-person representations also spontaneously tracked others’ beliefs about object identity, which modulated their duration of search in the box. Therefore, in contrast with recent proposals (Apperly & Butterfill, 2009), we provide evidence suggesting that computing others’ beliefs involving aspectuality may occur spontaneously and emerge early in development. Another study using an interactive helping task did not find evidence in toddlers for understanding false beliefs involving aspectuality (Fizke et al., 2017). However, the helping task differs in many aspects from the current task. For example, the continuous measure in our task may be more sensitive to capture these phenomena than the binary assessment in the helping task. Additionally, in our study infants who failed to grasp aspectuality from a first-person perspective also showed no evidence of belief attribution, and such factors may overshadow children’s sociocognitive abilities in other tasks too. Infants’ limitations in belief attribution may therefore at least in part come from limitations of their developing cognitive systems used in first-person reasoning—a possibility also supported by the correlation between infants’ behavior on the baseline and belief trials.

The intuitive prediction is that when it comes to complex third-person inferences, giving infants training in a specific first-person inference would also have an effect on infants’ understanding of others’ beliefs involving the same domain. For instance, while infants younger than 11.5 months usually pay little attention to the objects’ color compared to their shape (Wilcox, 1999), training them with the importance of color information in object individuation may also facilitate their understanding that others may use color information for individuation. Whether such effects are indeed observable in various domains and how fast such transfers may occur is a matter for future studies.

The current results support the possibility that infants can entertain various types of belief contents. Experiment 1 involved tracking the presence of an object form the other’s perspective (Object Absent condition) and its features (Object Absent-Exchanged condition), and potentially the absence of an object from the other’s point of view (Object Present condition). In Experiment 2, infants’ behavior suggested that those infants who appreciate the dual identity of objects from their own perspective can also track beliefs that can only be represented via grasping the aspectuality of mental representations. Such sensitivity to the other person’s belief involving aspectuality are difficult to explain with minimal mindreading accounts such as encoding belief-like states or registrations, as these have been proposed to involve agent–object relations that cannot deal with identity mistakes (Butterfill & Apperly, 2013), thus indicating that the ability to encode beliefs proper (Perner et al., 2011; Rakoczy, 2017) may be present early on. We also see no obvious ways to explain infants’ behavior on Experiments 1 and 2 with alternative mechanisms that would originate from differences in procedure between the trial types (Heyes, 2014). In each condition, the Actor leaves in both trials, she is away for a short period of time, and comes back in both types of trials directly before her retrieval of the object, immediately followed by the measurement period. Arguably, these two events (the Actor’s departure and return) are of comparable saliency, as neither occur in familiarization, and both direct infants’ attention away briefly from the box, thus they would cause comparable distraction, if any. Any explanation pertaining to attentional effects (as described in Heyes, 2014) would therefore lead to conflicting predictions with regard to the potential type of interference (retroactive or proactive) caused by the timing of two events (the Actor’s departure and her arrival) in the two trial types (true and false belief). For example, a proactive interference caused by the agent’s departure would predict that infants in false belief forget the change that happened in her absence, but crucially, the very same process triggered by the agent’s return would cause infants in both conditions not to remember that the agent subsequently took out an object from the box, leading to unclear predictions concerning search times in the two trial types.^2^ A retroactive interference triggered by the agent’s return would predict that infants forget what happened in her absence in false belief (the critical manipulation of adding/removing/transforming the object), but the same process would also predict that in true belief due to her departure infants should forget the event that happened before (again, the critical manipulation of adding/removing/transforming the object), thus overall predicting no differences between the trial types. We see no a priori reason based on which one could predict which type of attentional bias should occur in the present paradigm and applying them together lead to the above contradictory predictions. Finally, any explanation pertaining to low-level nonmentalistic effects should have led to a bias manifesting in differences between trials in Experiment 2 in the nonpassing group as well, where we did not observe any differences. These considerations together make submentalizing explanations unlikely.

Infants may recruit different potential cognitive mechanisms for solving such tasks that are compatible with our findings. On one hand, Southgate (2020) argues that an altercentric bias can be realized via mechanisms that do not entail attributing a representation to the other but encode the state of affairs congruent with the other’s perspective as stronger than their own. At the same time, an altercentric influence is arguably also compatible with metarepresentations and belief attribution, where the other’s perspective receives a considerable weight and thus influences one’s own behavior. Our findings do not differentiate between these alternatives. While more converging evidence is needed to reach a verdict on the sophistication of infant ToM, the present study provides strong motivation for further investigations.

## GENERAL DISCUSSION

Two experiments asked whether 14-month-old infants show altercentric modulation in an active behavioral task. Results showed that infants searched longer in a box when another person believed it contains an object, compared to when he or she believed that all had been retrieved. Infants’ own knowledge was identical in the two trials, therefore, the observed differences should be due to the other person’s belief about the content of the box.

This altercentric modulation is consistent with other studies showing that infants’ (Kovács et al., 2010) and adults’ (Freundlieb et al., 2016; Samson et al., 2010) behavior can be influenced by another agent’s belief or visual perspective. However, it also extends previous findings in two significant ways. First, the present scenarios are novel in that successful belief attribution required individuating and tracking multiple objects, and their appearance or identity. The current results therefore support the possibility that from early on infants can entertain various types of belief contents, including beliefs that involve appreciating the aspectuality of mental representations. Second, we found altercentric modulation in an active everyday behavior (searching) in early infancy, at an age at which, to our knowledge, there are no published reports documenting such effects. While looking time results (Kovács et al., 2010) indicate that infants’ attention can be modulated by others’ perspective, it was an open question whether they also manifest in infants’ consequent action planning. The fact that altercentrism affects infants’ active behavior speaks to the possibility that the phenomenon documented here is not simply a local, attention-directing mechanism but seems to be integrated into further processes, for instance, in those that control infants’ actions (Rakoczy, 2012).

While these results suggest that infants encoded the other person’s belief, at the same time they also open up questions regarding the relation between infants’ own representations and those of the Actor. While there was an effect of the other person’s belief (infants searched longer when the Actor believed an object remained in the box than when she believed it to be empty), there were seemingly no effects of the infants’ own beliefs between conditions in Experiment 1, as there was no overall difference between the conditions where an object was present and where no objects were present, though this may be in part due the lack of power for between-subjects comparisons in our sample. However, the present paradigm is not optimally suitable for such comparisons, as search times reflect a continuous measure of infants’ motivation to search, based on their own representation and that of the other. Differences in search time therefore show an influence of the other’s perspective, affecting infants’ response that is based on their own representation—therefore not necessarily overriding infants’ own representations but rather modulating them. Similar effects can sometimes be observed when after we learn some information, which later becomes discredited as it turns out to be a misinformation, it may nevertheless continue to influence our judgments (Ecker et al., 2010). As such, the current study was not designed directly to pit against each other infants’ own representations vs. those they form based on the other’s perspective (Southgate, 2020). We suggest that this question can be more suitably probed by designs where infants are presented with a binary choice, for example, to retrieve an object from location A or B, and assessed whether they select the location corresponding to their own memory or that of the other person. Recently, Kovács and colleagues (Kovács et al., 2021) found that infants search for an object where another person believes it to be if they themselves are not knowledgeable about the actual location of the object. A strong prediction would be that young infants may search for the object where the other person believes the object to be even if they had seen it to be moved elsewhere. The current results indicate that infants may be particularly prone to attend to another person’s perspective, and consequently the other’s belief affects their behavior to the extent that it might even outweigh their own representations (Southgate, 2020). An intriguing question remains how first-person and third-person (attributed) representations are separated, and how altercentric modulation may change over development.

In adults, altercentric effects are typically considered akin to a byproduct of our generic heightened sociality or an indication of spontaneous perspective taking, likely facilitating interpersonal coordination (Gallotti & Frith, 2013). From a developmental perspective, however, it could be advantageous to have a disposition to attend to others’ point of view from early on (Kampis et al., 2013; Southgate, 2020). Infants face a crucial learning problem, and the social environment is a useful source to select the most relevant sources of information. On one hand, attention to others’ perspective and beliefs would allow infants to learn *about* others, enabling infants to take part in social interactions with increasing success, via action coordination, action interpretation, and action prediction. On the other hand, such a sensitivity would also allow infants to learn about the material world *through the lenses of others* (Kampis et al., 2013). Kampis et al. (2013) found that 10-month-olds readily applied a preference they learned about one agent (preferring one object over another) also to another, novel agent; and argued that infants have treated the content of the other’s preference as universally applicable, generic information attached to the object. Thus, sensitivity to others’ mental states may serve a dual function early in human ontogeny, enabling infants both to learn about others, as well as to learn about the world via the perspective of others around them.

While altercentrism in general may serve a learning function by attuning infants to take others’ point of view, in the current studies it led to a seemingly suboptimal behavior: infants tended to search more even though they had reasons to believe there is no object present, and search less when there was in fact an object remaining, due to the influence of the other person’s perspective. As infants are typically surrounded by individuals who are more knowledgeable than themselves, a sensitivity to their perspective is likely to be generally advantageous. Such a disposition, however, can also manifest in signature biases such as ones observed in the false belief scenarios in the current study. Even in adults, altercentric influence can appear both as interference and as facilitatory with regard to one’s immediate task or goals. Sometimes we experience interference or intrusion where our judgment slows down or is more error-prone due to the influence of the other’s perspective (Elekes, Varga, & Király, 2016; Freundlieb et al., 2018; Surtees et al., 2016). Other times it has facilitatory effects when we observe something together with others, increasing our perceptual sensitivity, enabling faster detection of objects that appear in our environment, or leading to better remembering of items (He et al., 2011; Kovács et al., 2010; Seow & Fleming, 2019). It is an open question whether infants in our study could inhibit the other’s influence, for example, if they had a strong motivation to focus on their own perspective only. This could be probed by making the objects highly rewarding, or searching effortful, or by introducing alternative activities infants may choose to do instead of attending to the box. On the other hand, reducing the influence of the other may also be related to the capacity for self-other distinction. For example, adults show less interference in an imitation inhibition task when they are primed with self-related words or look in the mirror prior to the task (Spengler et al., 2010), indicating that a heightened focus on ourselves may make us less prone to altercentric interference. In a learning context, knowing when *not* to take over a piece of information from others is just as essential as being attuned to them. It is an open question whether an increased focus on themselves can be elicited in infants similarly as in adults, and whether it would lead to less influence of others’ perspective on their subsequent behavior.

A privileged status of others’ perspective in infancy also raises the question how infants select who to attend to, for example, in scenarios with multiple “others” with differing perspectives. We suggest that it is related to the broader subject of whose perspective and beliefs people track as well as to other domains of social cognition regarding whose actions we predict or whose goals we keep track of. There, too, likely there are selection mechanisms controlling whom we spend mental resources on in our environment. For infants, an important one is likely the relative relevance of those present around them (for a discussion, see also Southgate, 2020). For example, they are probably more likely to track the perspective of an interaction partner than a stranger or a bystander. Whether the partner is an in-group or out-group member may also influence altercentric effects on one’s judgments (Simpson & Todd, 2017), and altercentric effects are reduced when the other differs in age from the respondent (Ferguson et al., 2018). The current experiments implemented a design in which the main experimenter (E1/Actor) interacted the most with the child, and she also hid and retrieved the objects, making tracking her mental state about these objects relevant. The Confederate was present throughout, equally familiar but was passively sitting at the table. In many false belief tasks where there are multiple agents (and the caretaker is also typically present), there is a similar issue: either it is not as costly to track multiple perspectives, or studies have (perhaps unknowingly) managed to implement dynamics that make participants focus on one agent over another. Finally, another factor determining whose perspective infants attend to may be whose perspective differs from the infant’s. In the present study, the perspective of the other experimenter (E2) and that of the caregiver was congruent with the infant’s own perspective, therefore would not differentiate the critical trials. It is possible that a perspective that is identical with that of the infant may not be encoded as a belief (but rather as knowledge; see e.g., Dudley & Kovács, 2021; Phillips et al., 2021) and may not result in similar effects. It may also be that in case there are multiple other perspectives in a scene, the different perspectives of others may be privileged over the identical ones.

How handling multiple other-perspectives is achieved is underexplored even in adults. Some have found no altercentric effects when people observed multiple avatars with consistent or inconsistent perspective to each other, but always inconsistent with the participant’s perspective (Capozzi et al., 2014). Egocentric mistakes, however, remained—suggesting that there was some self-other interference even when two others’ perspectives were inconsistent with each other. An intriguing, related question is how to characterize handling self vs. other perspective and the relation between egocentric and altercentric cognition. While seemingly contradictory processes, egocentric and altercentric influence may not be mutually exclusive. Within the same scenario, one’s own point of view can influence how we make judgment of others’ perspective and vice versa; and depending on the context their relative weight, or which is easier to compute, may change (Kampis & Southgate, 2020, Box 4).

Further questions for future work remain whether altercentric effects would generalize to a variety of different populations (e.g., non-WEIRD societies, Henrich et al., 2010). Relatedly, the phylogenetic origins of altercentrism have yet to be uncovered. Recent evidence indicates that nonhuman apes and monkeys predict others’ actions based on their beliefs (Hayashi et al., 2020; Krupenye et al., 2016), and consequently some proposals have drawn strong parallels between apes’ and human infants’ sociocognitive abilities (Apperly & Butterfill, 2009; Tomasello, 2018). Yet, at least two aspects of the current findings suggest that such conclusions may be premature, as to our knowledge no altercentric effects have been demonstrated in nonhuman animals (Martin & Santos, 2014), and there is no evidence for the understanding of aspectuality of beliefs in any other species. The fact that human infants adopt the other’s perspective so readily points to a strong tendency to spontaneously focus on the other’s mental states and raises the crucial question whether the altercentric tendency of humans may be a species-unique phenomenon.

## ACKNOWLEDGMENTS

The authors thank the participating families; research assistants Borbála Köllőd, Eszter Körtvélyesi, Szilvia Takács, Iulia Savos, Karap Zsuzsanna, and others at the Cognitive Development Center, CEU, for their help with the many hours of data collection; Ildikó Király, Mikołaj Hernik, Gergely Csibra, and Victoria Southgate for discussions and input on earlier versions of the manuscript; and two reviewers whose constructive comments helped us improve this paper. This publication is the result of research conducted for Central European University, Private University – CEU GmbH. It was made possible by the CEU Open Access Fund.

## FUNDING INFORMATION

AMK, FP7 Ideas: European Research Council (https://dx.doi.org/10.13039/100011199), Award ID: 284236-REPCOLLAB. AMK, James S. McDonnell Foundation (https://dx.doi.org/10.13039/100000913), Award ID: 220020449.

## AUTHOR CONTRIBUTIONS

DK: Conceptualization: Equal; Formal analysis: Lead; Methodology: Lead; Visualization: Lead; Writing - Original Draft: Lead; Writing - Review & Editing: Equal. AMK: Conceptualization: Equal; Formal analysis: Supporting; Methodology: Supporting; Visualization: Supporting; Writing - Original Draft: Supporting; Writing - Review & Editing: Equal, Supervision: Lead.

## Notes

^1^ The minimum difference of 500 ms was a preset value aimed to approximate the shortest meaningful duration. Since search duration was coded from the moment when the child’s hand was inserted halfway into the box, until the moment when it was halfway taken out, this duration arguably reflects an initiated reaching/searching action. Importantly, however, the results are independent of whether we use the above-defined criteria that are derived from Experiment 1, or conceptually similar criteria derived from earlier studies (see the Supplemental Materials).^2^ Since in both conditions at least one object would remain according to infants’ memory, arguably in both trials they would have reasons to search. To our knowledge all versions of the search task in the literature used a comparison between 1 and 0 objects, as it is unclear whether infants should search longer if two objects are hidden or one. As such, this explanation leads to at least uncertain predictions, but most likely null findings between the two trial types.

## Supplemental Materials

https://doi.org/10.7910/DVN/ZVEBZH

## References

[bib1] Allen, M., Poggiali, D., Whitaker, K., Marshall, T. R., & Kievit, R. (2018). Raincloud plots: A multi-platform tool for robust data visualization. PeerJ Preprints. 10.7287/peerj.preprints.27137v1 PMC6480976 31069261

[bib2] Apperly, I. A., & Butterfill, S. A. (2009). Do humans have two systems to track beliefs and belief-like states? Psychological Review, 116(4), 953–970. 10.1037/a0016923, 19839692

[bib3] Baillargeon, R., Buttelmann, D., & Southgate, V. (2018a). Invited commentary: Interpreting failed replications of early false-belief findings: Methodological and theoretical considerations. Cognitive Development, 46(May), 112–124. 10.1016/j.cogdev.2018.06.001

[bib4] Baillargeon, R., Buttelmann, D., & Southgate, V. (2018b). Invited commentary: Interpreting failed replications of early false-belief findings: Methodological and theoretical considerations. Cognitive Development, 46(May), 112–124. 10.1016/j.cogdev.2018.06.001

[bib5] Baillargeon, R., Scott, R. M., & He, Z. (2010). False-belief understanding in infants. Trends in Cognitive Sciences, 14(3), 110–118. 10.1016/j.tics.2009.12.006, 20106714 PMC2930901

[bib6] Baldwin, D. A. (1991). Infants’ contribution to the achievement of joint reference. Child Development, 62(5), 875–890. 10.1111/j.1467-8624.1991.tb01577.x, 1756664

[bib7] Barone, P., Corradi, G., & Gomila, A. (2019). Infants’ performance in spontaneous-response false belief tasks: A review and meta-analysis. Infant Behavior and Development, 57(February), Article 101350. 10.1016/j.infbeh.2019.101350, 31445431

[bib8] Birch, S. A. J., & Bloom, P. (2004). Understanding children’s and adults’ limitations in mental state reasoning. Trends in Cognitive Sciences, 8(6), 255–260. 10.1016/j.tics.2004.04.011, 15165550

[bib9] Buttelmann, F., & Buttelmann, D. (2017). The influence of a bystander agent’s beliefs on children’s and adults’ decision-making process. Journal of Experimental Child Psychology, 153, 126–139. 10.1016/j.jecp.2016.09.006, 27741442

[bib10] Buttelmann, F., Suhrke, J., & Buttelmann, D. (2015). What you get is what you believe: Eighteen-month-olds demonstrate belief understanding in an unexpected-identity task. Journal of Experimental Child Psychology, 131, 94–103. 10.1016/j.jecp.2014.11.009, 25544393

[bib11] Butterfill, S. A., & Apperly, I. A. (2013). How to construct a minimal theory of mind. Mind and Language, 28(5), 606–637. 10.1111/mila.12036

[bib12] Cacchione, T., Schaub, S., & Rakoczy, H. (2013). Fourteen-month-old infants infer the continuous identity of objects on the basis of nonvisible causal properties. Developmental Psychology, 49(7), 1325–1329. 10.1037/a0029746, 22906060

[bib13] Capozzi, F., Cavallo, A., Furlanetto, T., & Becchio, C. (2014). Altercentric intrusions from multiple perspectives: Beyond dyads. PLoS ONE, 9(12), 1–14. 10.1371/journal.pone.0114210, 25436911 PMC4250177

[bib14] Carruthers, P. (2013). Mindreading in infancy. Mind & Language, 28(2), 141–172. 10.1111/mila.12014

[bib15] Dudley, R., & Kovács, Á. (2021). Do “knowledge attributions” involve metarepresentation just like belief attributions do? Behavioral and Brain Sciences, 44, Article E149. 10.1017/S0140525X20001594, 34796829

[bib16] Ecker, U. K. H., Lewandowsky, S., & Tang, D. T. W. (2010). Explicit warnings reduce but do not eliminate the continued influence of misinformation. Memory and Cognition, 38(8), 1087–1100. 10.3758/MC.38.8.1087, 21156872

[bib17] Egyed, K., Király, I., & Gergely, G. (2013). Communicating shared knowledge in infancy. Psychological Science, 24, 1348–1353. 10.1177/0956797612471952, 23719664

[bib18] el Kaddouri, R., Bardi, L., de Bremaeker, D., Brass, M., & Wiersema, J. R. (2019). Measuring spontaneous mentalizing with a ball detection task: putting the attention-check hypothesis by Phillips and colleagues (2015) to the test. Psychological Research, 84, 1749–1757. 10.1007/s00426-019-01181-7, 30976921

[bib19] Elekes, F., Bródy, G., Halász, E., & Király, I. (2016). Enhanced encoding of the co-actor’s target stimuli during a shared non-motor task. Quarterly Journal of Experimental Psychology, 69(12), 2376–2389. 10.1080/17470218.2015.1120332, 26624868

[bib20] Elekes, F., Varga, M., & Király, I. (2016). Evidence for spontaneous level-2 perspective taking in adults. Consciousness and Cognition, 41, 93–103. 10.1016/j.concog.2016.02.010, 26897297

[bib21] Elekes, F., Varga, M., & Király, I. (2017). Level-2 perspectives computed quickly and spontaneously: Evidence from eight- to 9.5-year-old children. British Journal of Developmental Psychology, 35(4), 609–622. 10.1111/bjdp.12201, 28833301

[bib22] Feigenson, L., & Carey, S. (2003). Tracking individuals via object-files: Evidence from infants’ manual search. Developmental Science, 6(5), 568–584. 10.1111/1467-7687.00313

[bib23] Feigenson, L., & Halberda, J. (2008). Conceptual knowledge increases infants’ memory capacity. Proceedings of the National Academy of Sciences of the United States of America, 105(29), 9926–9930. 10.1073/pnas.0709884105, 18626025 PMC2464616

[bib24] Ferguson, H. J., Brunsdon, V. E. A., & Bradford, E. E. F. (2018). Age of avatar modulates the altercentric bias in a visual perspective-taking task: ERP and behavioral evidence. Cognitive, Affective and Behavioral Neuroscience, 18(6), 1298–1319. 10.3758/s13415-018-0641-1, 30242574 PMC6244738

[bib25] Fizke, E., Butterfill, S., van de Loo, L., Reindl, E., & Rakoczy, H. (2017). Are there signature limits in early theory of mind? Journal of Experimental Child Psychology, 162, 209–224. 10.1016/j.jecp.2017.05.005, 28623778

[bib26] Freundlieb, M., Kovács, Á. M., & Sebanz, N. (2016). When do humans spontaneously adopt another’s visuospatial perspective? Journal of Experimental Psychology: Human Perception and Performance, 42(3), 401–412. 10.1037/xhp0000153, 26480249

[bib27] Freundlieb, M., Kovács, Á. M., & Sebanz, N. (2018). Reading your mind while you are reading—Evidence for spontaneous visuospatial perspective taking during a semantic categorization task. Psychological Science, 29(4), 614–622. 10.1177/0956797617740973, 29447070

[bib28] Gallotti, M., & Frith, C. D. (2013). Social cognition in the we-mode. Trends in Cognitive Sciences, 17(4), 160–165. 10.1016/j.tics.2013.02.002, 23499335

[bib29] Hanna, J. E., Tanenhaus, M. K., & Trueswell, J. C. (2003). The effects of common ground and perspective on domains of referential interpretation. Journal of Memory and Language, 49(1), 43–61. 10.1016/S0749-596X(03)00022-6

[bib30] Hayashi, T., Akikawa, R., Kawasaki, K., Egawa, J., Minamimoto, T., Kobayashi, K., Kato, S., Hori, Y., Nagai, Y., Iijima, A., Someya, T., & Hasegawa, I. (2020). Macaques exhibit implicit gaze bias anticipating others’ false-belief-driven actions via medial prefrontal cortex. Cell Reports, 30(13), 4433–4444.e5. 10.1016/j.celrep.2020.03.013, 32234478

[bib31] He, X., Lever, A. G., & Humphreys, G. W. (2011). Interpersonal memory-based guidance of attention is reduced for ingroup members. Experimental Brain Research, 211(3–4), 429–438. 10.1007/s00221-011-2698-8, 21519911

[bib32] Henrich, J., Heine, S. J., & Norenzayan, A. (2010). The weirdest people in the world? Behavioral and Brain Sciences, 33(2–3), 61–83. 10.1017/S0140525X0999152X, 20550733

[bib33] Herrmann, E., Call, J., Hernàndez-Lloreda, M. V., Hare, B., & Tomasello, M. (2007). Humans have evolved specialized skills of social cognition: The cultural intelligence hypothesis. Science, 317(5843), 1360–1366. 10.1126/science.1146282, 17823346

[bib34] Heyes, C. (2014). False belief in infancy: A fresh look. Developmental Science, 1–13. 10.1111/desc.12148, 24666559

[bib35] Kampis, D., Buttelmann, F., & Kovács, Á. M. (2020). Developing a theory of mind: Are infants sensitive to how other people represent the world? In The social brain: A developmental perspective (Issue 1978, pp. 143–160). MIT Press.

[bib36] Kampis, D., Parise, E., Csibra, G., & Kovács, Á. M. (2015). Neural signatures for sustaining object representations attributed to others in preverbal human infants. Proceedings of the Royal Society B: Biological Sciences, 282(1819), 0–1. 10.1098/rspb.2015.1683, 26559949 PMC4685805

[bib37] Kampis, D., Somogyi, E., Itakura, S., & Király, I. (2013). Do infants bind mental states to agents? Cognition, 129(2), 232–240. 10.1016/j.cognition.2013.07.004, 23942349

[bib38] Kampis, D., & Southgate, V. (2020). Altercentric cognition: How others influence our cognitive processing. Trends in Cognitive Sciences, 24(11), 944–959. 10.1016/j.tics.2020.09.003, 32981846

[bib39] Keysar, B., Lin, S., & Barr, D. J. (2003). Limits on theory of mind use in adults. Cognition, 89(1), 25–41. 10.1016/S0010-0277(03)00064-7 12893123

[bib40] Király, I., Oláh, K., Csibra, G., & Kovács, Á. M. (2018). Retrospective attribution of false beliefs in 3-year-old children. Proceedings of the National Academy of Sciences, 115(45), 11477–11482. 10.1073/pnas.1803505115, 30322932 PMC6233147

[bib41] Kovács, Á. M., Téglás, E., & Csibra, G. (2021). Can infants adopt underspecified contents into attributed beliefs? Representational prerequisites of theory of mind. Cognition. 10.1016/j.cognition.2021.104640, 33757642

[bib42] Kovács, Á. M., Téglás, E., & Endress, A. D. (2010). The social sense: Susceptibility to others’ beliefs in human infants and adults. Science, 330(6012), 1830–1834. 10.1126/science.1190792, 21205671

[bib43] Krupenye, C., Kano, F., Hirata, S., Call, J., & Tomasello, M. (2016). Great apes anticipate that other individuals will act according to false beliefs. Science, 354(6308), 110–114. 10.1126/science.aaf8110, 27846501

[bib44] Kulke, L., & Rakoczy, H. (2018). Implicit Theory of Mind—An overview of current replications and non-replications. Data in Brief, 16, 101–104. 10.1016/j.dib.2017.11.016, 29188228 PMC5694957

[bib45] Martin, A., & Santos, L. R. (2014). The origins of belief representation: Monkeys fail to automatically represent others’ beliefs. Cognition, 130(3), 300–308. 10.1016/j.cognition.2013.11.016, 24374209 PMC3963482

[bib46] Milward, S. J., Kita, S., & Apperly, I. A. (2014). The development of co-representation effects in a joint task: Do children represent a co-actor? Cognition, 132(3), 269–279. 10.1016/j.cognition.2014.04.008, 24853630

[bib47] Nickerson, R. S. (1999). How we know—And sometimes misjudge—What others know: Imputing one’s own knowledge to others. Psychological Bulletin, 125(6), 737–759. 10.1037/0033-2909.125.6.737

[bib48] Perner, J., Mauer, M. C., & Hildenbrand, M. (2011). Identity: Key to children’s understanding of belief. Science, 333(6041), 474–477. 10.1126/science.1201216, 21778403

[bib49] Perner, J., & Ruffman, T. (2005). Infants’ insight into the mind: How deep? Science, 308(5719), 214–216. 10.1126/science.1111656, 15821079

[bib80] Phillips, J., Buckwalter, W., Cushman, F., Friedman, O., Martin, A., Turri, J., Santos, L., & Knobe, J. (2021). Knowledge before belief. Behavioral and Brain Sciences, 44, e140. 10.1017/S0140525X20000618, 32895070

[bib50] Phillips, J., Ong, D. C., Surtees, A. D. R., Xin, Y., Williams, S., Saxe, R., & Frank, M. C. (2015). A second look at automatic theory of mind: Reconsidering Kovács, Téglás, and Endress (2010). Psychological Science, 26(9), 1353–1367. 10.1177/0956797614558717, 26253550

[bib51] Piaget, J. (1926). The language and thought of the children. Turbner and Co., Ltd.

[bib52] Poulin-Dubois, D., Rakoczy, H., Burnside, K., Crivello, C., Dörrenberg, S., Edwards, K., Krist, H., Kulke, L., Liszkowski, U., Low, J., Perner, J., Powell, L., Priewasser, B., Rafetseder, E., & Ruffman, T. (2018). Do infants understand false beliefs? We don’t know yet—A commentary on Baillargeon, Buttelmann and Southgate’s commentary. Cognitive Development, 48(November), 302–315. 10.1016/j.cogdev.2018.09.005

[bib53] Rakoczy, H. (2012). Do infants have a theory of mind? The British Journal of Developmental Psychology, 30(Pt 1), 59–74. 10.1111/j.2044-835X.2011.02061.x, 22429033

[bib54] Rakoczy, H. (2017). In defense of a developmental dogma: Children acquire propositional attitude folk psychology around age 4. Synthese, 194, 689–707. 10.1007/s11229-015-0860-8

[bib55] Samson, D., Apperly, I. A., Braithwaite, J. J., Andrews, B. J., & Bodley Scott, S. E. (2010). Seeing it their way: Evidence for rapid and involuntary computation of what other people see. Journal of Experimental Psychology: Human Perception and Performance, 36(5), 1255–1266. 10.1037/a0018729, 20731512

[bib56] Schneider, D., Bayliss, A. P., Becker, S. I., & Dux, P. E. (2012). Eye movements reveal sustained implicit processing of others’ mental states. Journal of Experimental Psychology: General, 141(3), 433–438. 10.1037/a0025458, 21910557

[bib57] Schober, M. F. (1993). Spatial perspective-taking in conversation. Cognition, 47(1), 1–24. 10.1016/0010-0277(93)90060-9, 8482069

[bib58] Schuwerk, T., Kampis, D., Baillargeon, R., Biro, S., Bohn, M., Byers-Heinlein, K., Dörrenberg, S., Fisher, C., Franchin, L., Fulcher, T., Garbisch, I., Geraci, A., Grosse Wiesmann, C. G., Hamlin, K., Haun, D. B. M., Hepach, R., Hunnius, S., Hyde, D. C., Karman, P., … Rakoczy, H. (2021, February 14). Action anticipation based on an agent’s epistemic state in toddlers and adults. PsyArXiv. 10.31234/osf.io/x4jbm

[bib59] Scott, R. M., & Baillargeon, R. (2009). Which penguin is this? Attributing false beliefs about object 18 months. Child Development, 80(4), 1172–1196. 10.1111/j.1467-8624.2009.01324.x, 19630901 PMC2965529

[bib60] Seow, T., & Fleming, S. M. (2019). Perceptual sensitivity is modulated by what others can see. Attention, Perception, and Psychophysics, 81(6), 1979–1990. 10.3758/s13414-019-01724-5, 31062300 PMC6675914

[bib61] Simpson, A. J., & Todd, A. R. (2017). Intergroup visual perspective-taking: Shared group membership impairs self-perspective inhibition but may facilitate perspective calculation. Cognition, 166, 371–381. 10.1016/j.cognition.2017.06.003, 28605699

[bib62] Southgate, V. (2020). Are infants altercentric? The Other and the Self in early social cognition. Psychological Review, 127(4), 505–523. 10.1037/rev0000182, 31868391

[bib63] Southgate, V., & Vernetti, A. (2014). Belief-based action prediction in preverbal infants. Cognition, 130(1), 1–10. 10.1016/j.cognition.2013.08.008, 24140991 PMC3857687

[bib64] Spengler, S., Brass, M., Kühn, S., & Schütz-Bosbach, S. (2010). Minimizing motor mimicry by myself: Self-focus enhances online action-control mechanisms during motor contagion. Consciousness and Cognition, 19(1), 98–106. 10.1016/j.concog.2009.12.014, 20116291

[bib65] Stahl, A. E., & Feigenson, L. (2014). Social knowledge facilitates chunking in infancy. Child Development, 85(4), 1477–1490. 10.1111/cdev.12217, 24433226

[bib66] Stahl, A. E., & Feigenson, L. (2018). Infants use linguistic group distinctions to chunk items in memory. Journal of Experimental Child Psychology, 172, 149–167. 10.1016/j.jecp.2018.03.005, 29626755

[bib67] Surtees, A., Apperly, I., & Samson, D. (2016). I’ve got your number: Spontaneous perspective-taking in an interactive task. Cognition, 150, 43–52. 10.1016/j.cognition.2016.01.014, 26848735

[bib68] Thoermer, C., Sodian, B., Vuori, M., Perst, H., & Kristen, S. (2012). Continuity from an implicit to an explicit understanding of false belief from infancy to preschool age. The British Journal of Developmental Psychology, 30(Pt 1), 172–187. 10.1111/j.2044-835X.2011.02067.x, 22429040

[bib69] Tomasello, M. (2018). How children come to understand false beliefs: A shared intentionality account. Proceedings of the National Academy of Sciences, 115(34), 8491–8498. 10.1073/pnas.1804761115, 30104372 PMC6112688

[bib70] Van de Walle, G. A., Carey, S., & Prevor, M. (2000). Bases for object individuation in infancy: Evidence from manual search. Journal of Cognition and Development, 1(3), 249–280. 10.1207/S15327647JCD0103_1

[bib71] Van Der Wel, R. P. R. D., Sebanz, N., & Knoblich, G. (2014). Do people automatically track others’ beliefs? Evidence from a continuous measure. Cognition, 130(1), 128–133. 10.1016/j.cognition.2013.10.004, 24216021

[bib72] Wilcox, T. (1999). Object individuation: Infants’ use of shape, size, pattern, and color. Cognition, 72(2), 125–166. 10.1016/S0010-0277(99)00035-9, 10553669

